# Q & A: The Alzheimer's disease neuroimaging initiative

**DOI:** 10.1186/1741-7015-9-101

**Published:** 2011-09-01

**Authors:** Paul S Aisen

**Affiliations:** 1 Department of Neurosciences University of California San Diego 9500 Gilman Drive, M/C 0949 La Jolla, CA 92093 USA

## What is the Alzheimer's Disease Neuroimaging Initiative and who funds it?

The Alzheimer's Disease Neuroimaging Initiative (ADNI) is a large, highly collaborative longitudinal cohort study aiming to elucidate biomarker trajectories in Alzheimer's disease (AD). ADNI was initially funded in 2004, based on the urgent need to understand potential biomarkers better in order to facilitate drug development, and the large cost and effort required to conduct appropriate studies. About two thirds of its budget is provided by the US National Institute on Aging (NIA), and about one third comes from pharmaceutical companies; private foundations have also contributed.

## Who participates in the initiative?

ADNI is led by Dr. Michael Weiner at UCSF. The core operations of the program reside at UCSD, UCLA, UCDavis, UC Berkeley, U Penn and the Mayo Clinic. In addition, there are more than 50 performance sites across the US and Canada.

ADNI investigators study structural and molecular imaging, blood and cerebrospinal fluid markers, and genetic markers in cohorts of older individuals followed longitudinally over years. The cohorts include cognitively normal individuals, as well as those with mild amnestic cognitive impairment and mild AD. Just over 800 individuals enrolled in the original ADNI cohorts; all received structural magnetic resonance imaging (MRI), cognitive, clinical and blood analyses; over half underwent lumbar punctures, and subgroups were studied by fluorodeoxyglucose positron emission tomography (FDG-PET) and amyloid PET imaging.

## What were the major achievements of the initiative in the first five years?

ADNI has been enormously successful, generating more than 200 scientific publications. Among its achievements are the standardization of imaging and biochemical biomarker methodologies, characterization of these biomarkers cross-sectionally and longitudinally across the cohorts, demonstration of the relationships among these diverse measures, and interpretation of its wealth of data in terms of biomarker models of AD.

ADNI methods and data are utilized by virtually all academic and commercial AD drug development research worldwide. ADNI has been launched in Japan, and aspects of ADNI are reflected in biomarker studies throughout Europe, Asia and Australia. The data have contributed substantially to recent revisions of diagnostic criteria for AD and mild cognitive impairment (MCI).

## Have these achievements been translated to the clinic?

While the impact of ADNI on the AD research community is inarguable, its aims are not directly related to clinical practice. The impact of ADNI on the clinic will be realized when its methods succeed in accelerating the development of effective disease-modifying treatments, the overarching aim of ADNI.

## Is funding a problem in the current climate?

We are fortunate that ADNI has been extended for a new five year period. ADNI-2, which recently started, has a funding structure quite similar to the original ADNI-1, with two thirds from NIA, and most of the rest from industry contributions. The ADNI team is greatly appreciative of this financial support despite the current economic pressures.

## What are the aims for ADNI-2?

ADNI-2 continues and extends the original aims of ADNI. The original cognitively normal and MCI cohorts continue to be followed, so that we can more fully elucidate the entire sequence of events from normal aging through dementia. A new, very mildly impaired group, called early MCI, has been defined and is being enrolled, along with additional individuals for each of the original cohorts; recent funding supports the enrollment of a total of 700 additional participants. Structural MRI, FDG-PET, amyloid PET, lumbar punctures, genetic analysis and blood studies, along with cognitive, behavioral and clinical assessments, are obtained on all participants. New amyloid PET and structural and functional MRI methods are now being employed. All data continue to be shared publicly as they are collected.

## How has ADNI contributed to your research?

My work, primarily through the Alzheimer's Disease Cooperative Study ((ADCS), another large consortium supported by NIA) aims to develop more effective treatments for AD. The ADCS conducts randomized controlled therapeutic trials. Our trial designs and methods utilize lessons from ADNI extensively.

We believe that the best path to effective disease-modifying treatment is to move our therapeutic interventions to the earliest possible stage of disease, perhaps even to the asymptomatic stage. ADNI data have provided the means of identifying individuals at such early stages and revealed outcome measures that will allow us to assess treatment effects in the absence of clinical symptoms. The value of ADNI data to our work cannot be overstated.

## What are the ways in which the experience gained by ADNI might be used by other areas?

Two aspects of ADNI deserve the attention of the wider scientific community: extensive collaboration and open data sharing. Much has been written about the conflicting aims of profit-driven pharmaceutical companies, academic investigators, regulatory bodies and patients and families. ADNI has demonstrated, beyond question, that the aims of these groups overlap, and that cooperation is enormously fruitful. All participate in the design and management of ADNI. And, crucially, all ADNI data are immediately shared openly with interested individuals worldwide; data are posted to the web daily, and shared at no cost to virtually all who request it. Dozens of ADNI papers come from investigators with no affiliation to the study. All companies, regardless of whether they contribute financially, have full access to the data.

The ADNI model of extensive collaboration and public data sharing should, and I think will, spread to and benefit other areas of science and medicine.

## Do you have any comments for patients or carers of AD patients?

The major message from ADNI to the community of patients and families affected by AD is one of increased hope. ADNI continues to substantially improve the odds that we will soon have much more effective treatments for this disease.

## Where can I find out more?

**Alzheimer's Disease Neuroimaging Initiative**, http://www.adni-info.org

**Alzheimer's Disease Cooperative Study**, http://adcs.org

Petersen RC, Aisen PS, Beckett LA, Donohue MC, Gamst AC, Harvey DJ, Jack CR Jr, Jagust WJ, Shaw LM, Toga AW, Trojanowski JQ, Weiner MW: **Alzheimer's Disease Neuroimaging Initiative (ADNI): clinical characterization. ***Neurology *2010, **74: **201-209.

Aisen PS, Petersen RC, Donohue M, Gamst A, Raman R, Thomas RG, Walter S, Trojanowski JQ, Shaw L, Beckett LA, Jack CR, Jagust W, Toga A, Saykin AJ, Green RC, Morris JC and MW Weiner for the Alzheimer's Disease Neuroimaging Initiative: **Clinical core of the Alzheimer's Disease Neuroimaging Initiative: progress and plans. ***Alzheimer's & Dementia *2010, **6:**239-246.

## Abbreviations

AD: Alzheimer's disease; ADNI: Alzheimer's Disease Neuroimaging Initiative; ADCS: Alzheimer's Disease Cooperative Study; FDG-PET: fluorodeoxyglucose positron emission tomography; MCI: mild cognitive impairment.

